# Addition of routine blood biomarkers to TIMI risk score improves predictive performance of 1-year mortality in patients with ST-segment elevation myocardial infarction

**DOI:** 10.1186/s12872-020-01777-7

**Published:** 2020-11-18

**Authors:** Pyung Chun Oh, Young Sil Eom, Jeonggeun Moon, Ho-Jun Jang, Tae-Hoon Kim, Jon Suh, Min Gyu Kong, Sang-Don Park, Sung Woo Kwon, Soon Yong Suh, Kyounghoon Lee, Seung Hwan Han, Taehoon Ahn, Woong Chol Kang

**Affiliations:** 1 Department of Cardiology, Gil Medical Center, Gachon University College of Medicine, 1198 Guwol-dong, Namdong-gu, Incheon, Republic of Korea 405-760; 2 Department of Endocrinology and Metabolism, Gil Medical Center, Gachon University College of Medicine, Incheon, Republic of Korea; 3 Department of Cardiology, Sejong General Hospital, Bucheon, Republic of Korea; 4 Department of Cardiology, Soon Chun Hyang University Bucheon Hospital, Bucheon, Republic of Korea; 5 Department of Cardiology, Inha University Hospital, Incheon, Republic of Korea

**Keywords:** ST-segment elevation myocardial infarction, Hypoxic liver injury, Dysglycemia, Anemia, Neutrophil to lymphocyte ratio, Risk score, Mortality

## Abstract

**Background:**

Several biomarkers have been proposed as independent predictors of poor outcomes in ST-segment elevation myocardial infarction (STEMI). We investigated whether adding information obtained from routine blood tests including hypoxic liver injury (HLI), dysglycemia, anemia, and high neutrophil to lymphocyte ratio (NLR) could improve the prognostic performance of the TIMI risk score for the prediction of 1-year mortality.

**Methods:**

A total of 1057 patients with STEMI undergoing primary percutaneous coronary intervention (PCI) between 2007 and 2014 were retrospectively enrolled from 4-regional hospitals. HLI and dysglycemia were defined as serum transaminase > twice the normal upper limit and glucose < 90 or > 250 mg/dL, respectively. The effect of adding biomarkers to the TIMI risk score on its discriminative ability was assessed using c-statistic, net reclassification improvement (NRI), and integrated discrimination improvement (IDI).

**Results:**

The 1-year mortality rate was 7.1%. The best cutoff value of NLR for the prediction of 1-year mortality was 4.3 (sensitivity, 67%; specificity, 65%). HLI (HR 2.019; 95% CI 1.104–3.695), dysglycemia (HR 2.535; 95% CI 1.324–3.923), anemia (HR 2.071; 95% CI 1.093–3.923), and high NLR (HR 3.651; 95% CI 1.927–6.918) were independent predictors of 1-year mortality. When these 4 parameters were added to the TIMI risk score, the c-statistic significantly improved from 0.841 to 0.876 (*p* < 0.001), and the NRI and IDI were estimated at 0.203 (95% CI 0.130–0.275; *p* < 0.001) and 0.089 (95% CI 0.060–0.119; *p* < 0.001), respectively.

**Conclusions:**

The addition of HLI, dysglycemia, anemia, and high NLR to the TIMI risk score may be useful for very early risk stratification in patients with STEMI receiving primary PCI.

## Background

Several risk stratification scores have been designed to predict mortality or complications in patients with non-ST-segment elevation myocardial infarction (NSTEMI) and ST-segment elevation myocardial infarction (STEMI). Recent guidelines recommend using risk scores, for early risk assessment and adjustment, such as the TIMI (Thrombolysis in Myocardial Infarction) for STEMI, TIMI for NSTEMI, and GRACE (Global Registry of Acute Coronary Events) for both types of myocardial infarction (MI) [1, 2].

Recent studies suggest that several biomarkers evaluated during routine blood tests upon admission may be independent predictors of poor clinical outcomes in patients with STEMI [3–5]. We previously demonstrated that the presence of hypoxic liver injury (HLI), defined as serum transaminase levels more than two times of the upper normal limit, was associated with all-cause mortality and major adverse cardiovascular events in STEMI patients [5]. Dysglycemia, defined as serum glucose < 90 or > 250 mg/dL at the time of presentation, was an independent predictor of in-hospital mortality in STEMI [6]. In addition, the neutrophil to lymphocyte ratio (NLR) has been demonstrated as a prognostic biomarker for various cardiovascular diseases [7, 8]. Several studies have revealed that STEMI patients with anemia had a higher risk of cardiovascular mortality [3, 9]. However, there was little evidence that examining these biomarkers provided any incremental value to conventional risk scoring models. Therefore, we investigated whether the addition of HLI, dysglycemia, NLR, and anemia, measured upon on admission, could improve the prognostic performance of the TIMI risk score for 1-year mortality in patients with STEMI who underwent primary percutaneous coronary intervention (PCI).

## Methods

### Study population

We retrospectively analyzed the cohort data from INTERSTELLAR (INcheon-Bucheon cohorT of patients undERgoing primary PCI for acute ST-ELevation myocardiaL infarction; clinicaltrials.gov identifier NCT02804958) [10, 11]. The INTERSTELLAR registry was retrospectively established by four hospitals in the Incheon-Bucheon province. This cohort consisted of 1540 consecutive patients with STEMI who underwent primary PCI between 2007 and 2014. The study protocol was approved by the Institutional Review Boards of the four participating hospitals and written informed consent was obtained from each patient. In this cohort, patients were excluded if they had any one of the following; primary cardiomyopathy, significant valvular heart disease (≥ moderate), pericardial disease, known liver disease, or history of potentially hepatotoxic medications within the past 3 months. Among these, all 483 patients from one hospital were excluded because their baseline laboratory data, particularly the NLR, was incomplete. Thus, 1057 patients from the remaining three hospitals were enrolled in this analysis. All procedures and in-hospital management were performed according to current standard guidelines, as previously described [11, 12].

### Definition of variables and endpoint

HLI was defined as serum transaminase levels of more than two times the normal upper limit (i.e., aspartate transaminase > 80 U/L or alanine transaminase > 80 U/L), as per Moon et al. [5]. We defined dysglycemia as serum glucose < 90 or > 250 mg/dL, based on our previous study using the same cohort [6]. In our previous study [6], the median serum glucose level of the overall population was 144 mg/dL. Among study subjects with serum glucose < 140 mg/dL in emergency room, glucose < 89.5 mg/dL was a highly specific predictor of in-hospital death (sensitivity 25%; specificity 97%; area under the curve [AUC] 0.683; *p* = 0.005). Similarly, among patients with serum glucose > 140 mg/dL, a cut-off value of 241.5 mg/dL predicted in-hospital death (sensitivity, 60%; specificity, 74%; AUC 0.706; *p* < 0.001). According to the World Health Organization classification, anemia was defined as a hemoglobin level of < 12.0 g/dL in women and < 13.0 g/dL in men. The best cut-off value for NLR was statistically calculated. The primary endpoint was cumulative all-cause death in 1 year. All medical records were reviewed to collect patient follow-up data. In the case of follow-up failure, standardized telephone interviews were conducted.

### Statistical analysis

Continuous data are presented as means ± standard deviations and/or medians (interquartile ranges), as appropriate. The categorical variables are described using percentage or absolute number. Baseline characteristics were compared across patients with and without 1-year all-cause mortality. A receiver operating characteristics (ROC) curve was calculated to determine the cut-off value of NLR for predicting 1-year mortality. The cut-off value for optimal sensitivity and specificity, together with the AUC, was also identified. Multivariate Cox regression analysis was performed to identify independent predictors of primary endpoint. In the full regression model, the hazard ratio for each of the additional blood biomarkers was calculated. For each patient, additional risk scores were calculated as the simple arithmetic sum of point values assigned to each risk factor based on the multivariate-adjusted risk relationship: 1 point for hazard ratio (HR) 1.0–< 2.5, and 2 points for HR > 2.5. The discriminative ability and prognostic performance of the combination of TIMI risk score and blood biomarkers, as compared to the TIMI risk score alone, was assessed by C-statistics, integrated discrimination index (IDI), and net reclassification index (NRI). Risk categories for mortality were set at < 1% for low risk, 1%-5% for intermediate risk and > 5% for high risk. The calibration of models was assessed by the Hosmer and Lemeshow test. No adjustments for multiple testing were performed as this was an exploratory analysis. P values of less than 0.05 were considered statistically significant. The analysis was performed using SAS version 9.4 (SAS Institute, Cary, NC, USA).

## Results

### Baseline characteristics

The mean age was 60.5 ± 13.1 years, and 79.2% of the patients were men. Median duration of follow-up was 27 months (interquartile range, 10–41 months). One-year mortality was seen in 75 patients (7.1%). Table 1 shows the demographic and laboratory data measured at the time of presentation and intergroup comparison between the groups with or without 1-year mortality, defined henceforth as Mortality (+) or Mortality (−), respectively. Mortality (+) patients were significantly older and had lower blood pressure, higher pulse rate, lower body mass index, higher Killip class, and higher prevalence of anterior wall infarction. Regarding the laboratory findings, the Mortality (+) group showed significantly higher serum transaminases, lower albumin, higher glucose, lower estimated glomerular filtration rate, lower hemoglobin, higher white blood cell counts, and higher NLR. The HLI, dysglycemia, and anemia were significantly higher in the Mortality (+) group than in the Mortality (−) group (49.3 vs 20.3% for HLI, 44.0 vs. 12.8% for dysglycemia, 41.3 vs. 14.9% for anemia, respectively, all *p* < 0.001). For predicting 1-year mortality, the cut-off value of NLR was determined to be 4.3, with a sensitivity and specificity of 66.7% and 65.2%, respectively (AUC 0.681; 95% CI 0.614–0.749, *p* < 0.001). High NLR values (> 4.3) were more frequent in the Mortality (+) group than those in the Mortality (−) group (68.0 vs 34.8%, respectively, *p* < 0.001).

**Table 1:** Demographic and laboratory data

	All (n = 1057)	Mortality (−) (n = 982)	Mortality (+) (n = 75)	*p* value
Demographic data				
Age (years)	60.5 ± 13.1	59.7 ± 12.9	70.8 ± 11.0	< 0.001
Men [n (%)]	837 (79.2)	784 (79.8)	53 (70.7)	0.075
Body mass index (kg/m^2^)	24.1 ± 3.2	24.2 ± 3.2	23.0 ± 3.6	0.002
Diabetes mellitus [n (%)]	279 (26.4)	250 (25.5)	29 (38.7)	0.020
Hypertension [n (%)]	496 (46.9)	452 (46.0)	44 (58.7)	0.041
Systolic blood pressure (mmHg)	125.1 ± 27.3	126.3 ± 26.7	109.0 ± 30.8	< 0.001
Diastolic blood pressure (mmHg)	76.3 ± 17.5	77.0 ± 17.2	66.9 ± 19.2	< 0.001
Heart rate (beats/minute)	77.5 ± 20.1	76.7 ± 19.5	87.3 ± 25.0	0.001
Cardiogenic shock [n (%)]	66 (6.2)	47 (4.8)	19 (25.3)	< 0.001
Killip class II–IV [n (%)]	209 (19.8)	168 (17.1)	41 (54.7)	< 0.001
Anterior wall infarction [n (%)]	564 (69.3)	512 (52.1)	52 (69.3)	0.004
Laboratory data				
Albumin (g/dL)	4.2 ± 0.4	4.2 ± 0.4	3.8 ± 0.6	< 0.001
Glucose (mg/dL)	177.7 ± 83.1	173.0 ± 74.6	239.4 ± 144.0	< 0.001
Dysglycemia [n (%)]	159 (15.0)	126(12.8)	33 (44.0)	< 0.001
Total bilirubin (mg/dL)	0.7 ± 0.4	0.7 ± 0.4	0.7 ± 0.5	0.245
AST (IU/L)	33.0 (23.0–65.0)	32.0 (23.0–61.0)	72.0 (27.0–227.0)	< 0.001
ALT (IU/L)	26.0 (19.0–42.0)	26.0 (19.0–41.0)	36.0 (18.0–78.0)	0.034
Hypoxic liver injury^†^ [n (%)]	236 (22.3)	199 (20.3)	37 (49.3)	< 0.001
ALP (IU/L)	74.0 (61.0–91.1)	74.0 (61.0–90.0)	81.0 (65.0–98.0)	0.061
Creatinine (mg/dL)	1.08 ± 0.73	1.06 ± 0.73	1.33 ± 0.67	0.001
Estimated GFR (mL/min/1.73 m^2^)	82.1 ± 27.5	83.3 ± 26.8	65.8 ± 32.0	< 0.001
Estimated GFR < 60 mL/min/1.73 m^2^ [n (%)]	184 (17.4)	152 (15.5)	32 (42.7)	< 0.001
Hemoglobin (mg/dL)	14.2 ± 1.9	14.3 ± 1.9	13.1 ± 2.4	< 0.001
Anemia [n (%)]	177 (16.7)	146 (14.9)	31 (41.3)	< 0.001
WBC (× 10^3^/µL)	12.0 ± 5.2	11.8 ± 5.2	13.9 ± 4.7	0.001
NLR	3.1 (1.6–5.7)	3.0 (1.5–5.4)	5.4 (3.0–8.9)	< 0.001
NLR > 4.3 [n (%)]	393 (37.2)	342 (34.8)	51 (68.0)	< 0.001
Total cholesterol (mg/dL)	189.3 ± 43.7	190.6 ± 43.1	172.5 ± 47.8	0.001
LDL-cholesterol (mg/dL)	117.0 ± 36.8	117.8 ± 36.7	106.6 ± 37.0	0.043
HDL-cholesterol (mg/dL)	42.5 ± 11.0	42.6 ± 10.9	41.0 ± 11.7	0.305
Triglyceride	129.0 (86.0–196.0)	131.0 (87.0–197.0)	111.0 (76.5–180.5)	0.100
Initial CK-MB (ng/mL)	5.3 (2.1–26.5)	4.9 (2.1–22.5)	26.0 (3.6–96.1)	< 0.001
Peak CK-MB (ng/mL)	177.4 (78.2–300.0)	170.9 (77.8–300.0)	271.0 (86.0–332.2)	0.016

*AST* aspartate transaminase, *ALT* alanine transaminase, *ALP* alkaline phosphatase, *GFR* glomerular filtration rate, *WBC* white blood cell, *NLR* neutrophil to lymphocyte ratio, *CK-MB* creatine kinase-myocardial band isoenzyme

^*^Dysglycemia was defined as serum glucose < 90 or > 250 mg/dL

^†^Hypoxic liver injury was defined as an elevation of serum transaminases levels more than twice the upper limit of normal

Angiographic, procedural, and echocardiographic data is summarized in Table 2. Although baseline TIMI flow grade was similar between the two groups, the final TIMI flow grades and procedural success rates were significantly lower in the Mortality (+) group. The Mortality (+) group also had longer door-to-balloon time and symptom-to-balloon time. Further, the left ventricular ejection fraction was significantly lower in the Mortality (+) group than that in the Mortality (−) group (35.7 vs. 48.0%, respectively, *p* < 0.001).

**Table 2:** Angiographic, procedural and echocardiographic data

	All (n = 1057)	Mortality (−) (n = 982)	Mortality (+) (n = 75)	*p* value
Angiographic and procedural data				
Infarct related artery [n (%)]				NA
Left main	8 (0.8)	2 (0.2)	6 (8.0)	
Left anterior descending	556 (52.6)	510 (51.9)	46 (61.3)	
Left circumflex	95 (9.0)	90 (9.2)	5 (6.7)	
Right coronary	398 (37.7)	380 (38.7)	18 (24.0)	
Extent of coronary artery disease [n (%)]				0.257
1-vessel	404 (38.2)	382 (38.9)	22 (29.3)	
2-vessel	364 (34.4)	334 (34.0)	30 (40.0)	
3-vessel	289 (27.3)	266 (27.1)	23 (30.7)	
Baseline TIMI flow grade [n (%)]				0.840
0–2	950 (90.0)	884 (90.1)	66 (89.2)	
3	105 (10.0)	97 (9.9)	8 (10.8)	
Final TIMI flow grade [n (%)]				< 0.001
0–2	163 (15.5)	134 (13.7)	29 (39.2)	
3	892 (84.5)	847 (86.3)	45 (60.8)	
Stent number (n)	1.1 ± 0.3	1.1 ± 0.3	1.2 ± 0.5	0.036
Stent diameter (mm)	3.09 ± 0.38	3.10 ± 0.38	2.95 ± 0.40	0.002
Stent length (mm)	27.1 ± 10.5	26.8 ± 10.3	31.0 ± 13.3	0.012
Door-to-balloon time (min)	72.0 (58.0–87.0)	71.0 (58.0–87.0)	80.0 (64.5–89.0)	0.012
Symptom-to-balloon time (min)	210.0 (135.0–407.5)	206.0 (133.0–381.3)	296.0 (180.0–570.0)	0.001
Procedural success [n (%)]	892 (84.5)	847 (86.3)	45 (60.8)	< 0.001
Echocardiographic data				
LVEF (%)	47.3 ± 12.0	48.0 ± 11.5	35.7 ± 14.9	< 0.001
LVEDD (mm)	51.4 ± 5.0	51.4 ± 5.0	51.2 ± 4.8	0.897
E/E’	11.9 ± 5.2	11.8 ± 5.2	12.8 ± 4.7	0.564

*NA* not available, *TIMI* thrombolysis in myocardial infarction, *LVEF* left ventricular ejection fraction, *LVEDD* left ventricular end-diastolic dimension

### Predictors of 1-year mortality

In the multivariate Cox regression analysis, age, systolic blood pressure < 100 mmHg, Killp class II–IV, left ventricular ejection fraction < 40%, and anterior wall infarction were significant and were independent predictors for 1-year mortality (Table 3). Among the TIMI risk score variables, history of diabetes mellitus, hypertension, or angina, heart rate, body weight, and ischemia time were not significant risk factors in the multivariate analysis. However, HLI, dysglycemia, anemia, and high NLR were all independently associated with increased risk of 1-year mortality.

**Table 3:** Predictors for 1-year all-cause death

Variables	Univariate analysis			Multivariate analysis		
	HR	95% CI	*p* value	HR	95% CI	*p* value
Age (per 10-year-old increase)	1.960	1.621–2.370	< 0.001	1.416	1.097–1.828	0.008
Male	0.610	0.371–1.003	0.051	1.783	0.802–3.962	0.156
Body weight < 67 kg	1.807	1.123–2.907	0.015	1.555	0.735–3.291	0.249
Diabetes mellitus	1.824	1.146–2.903	0.011	1.062	0.496–2.272	0.878
Hypertension	1.655	1.045–2.621	0.032	0.970	0.489–1.923	0.930
Systolic BP < 100 mmHg	4.459	2.770–7.178	< 0.001	2.568	1.357–4.860	0.004
Heart rate > 100 beats/minute	3.022	1.796–5.085	< 0.001	1.239	0.548–2.802	0.606
Killip class II–IV	5.445	3.455–8.581	< 0.001	2.426	1.367–4.303	0.002
LVEF < 40%	5.080	2.981–8.658	< 0.001	1.889	1.008–3.541	0.047
Anterior wall infarction	2.061	1.261–3.366	0.004	2.084	1.056–4.114	0.034
Multivessel disease	1.526	0.928–2.509	0.095	1.047	0.511–2.144	0.900
Post-TIMI flow < 3	3.782	2.371–6.032	< 0.001	1.843	1.006–3.379	0.048
Hypoxic liver injury*	3.631	2.308–5.710	< 0.001	2.019	1.104–3.695	0.023
Dysglycemia^†^	5.008	3.173–7.904	< 0.001	2.535	1.324–4.855	0.005
Anemia	3.702	2.338–5.863	< 0.001	2.071	1.093–3.923	0.026
NLR > 4.3	3.796	2.336–6.166	< 0.001	3.651	1.927–6.918	< 0.001
Estimated GFR < 60 mL/min/1.73 m^2^	3.879	2.454–6.131	< 0.001	0.791	0.364–1.720	0.554
Peak CK-MB (log)	1.637	0.951–2.821	0.075	1.228	0.618–2.441	0.558
Symptom to balloon time > 4 h	2.094	1.316–3.333	0.002	1.477	0.731–2.988	0.277

*HR* hazard ratio, *CI* confidence interval, *BP* blood pressure, *LVEF* left ventricular ejection fraction, *NLR* neutrophil to lymphocyte ratio, *GFR* glomerular filtration rate, *CK-MB* creatine kinase-myocardial band isoenzyme, *ALP* alkaline phosphatase

^*^Hypoxic liver injury was defined as an elevation of serum transaminase level more than twice the upper limit of normal

^†^Dysglycemia was defined as serum glucose < 90 or > 250 mg/dL

### Prognostic value of HLI, dysglycemia, NLR, and anemia over the TIMI risk score

**Fig. 1:**
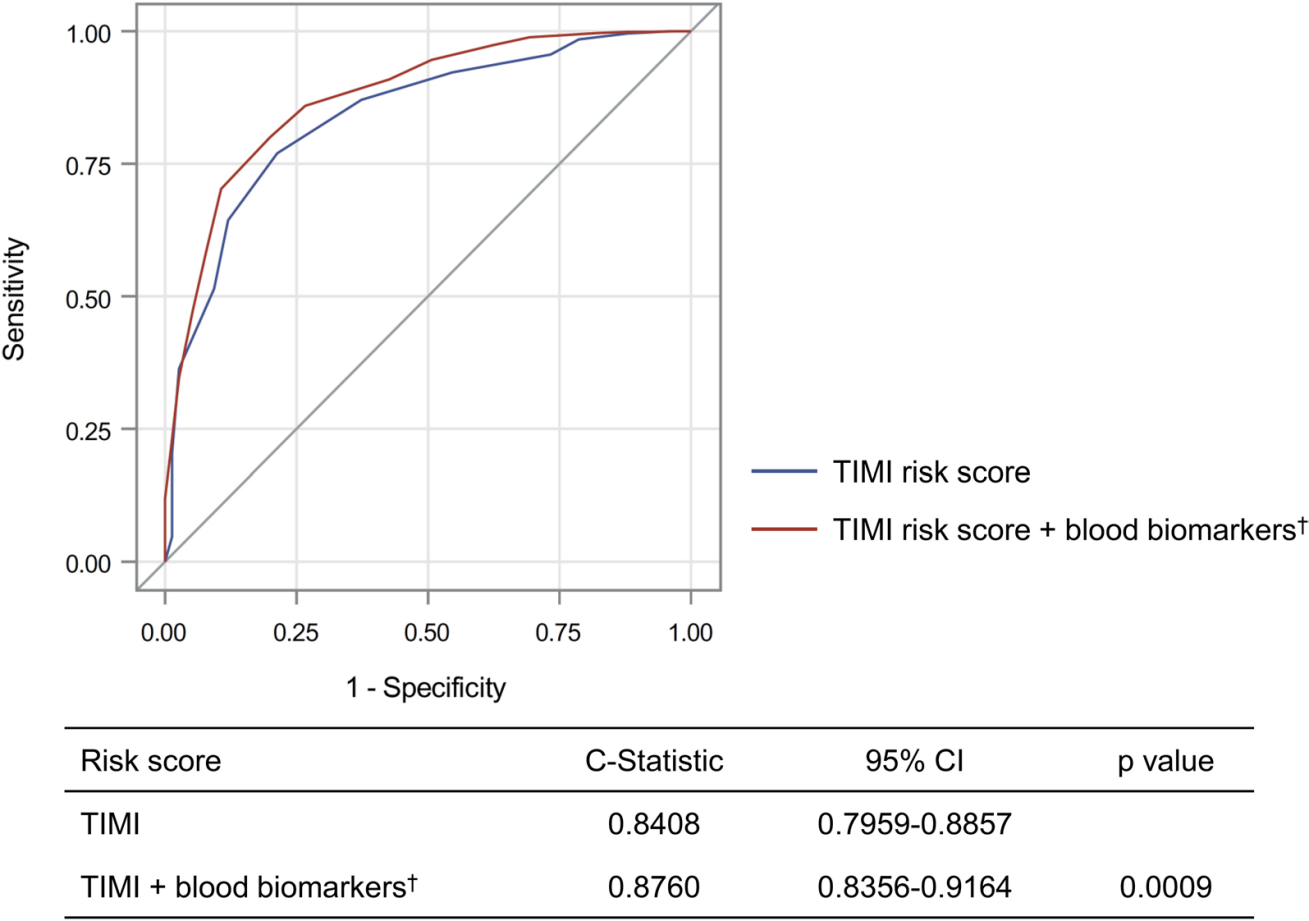
Receiver operating curves for the predicted probabilities of selected risk scores before (blue line) and after (red line) the addition of four biomarkers to the conventional TIMI risk score. ^†^The added biomarkers included hypoxic liver injury, dysglycemia, anemia and high neutrophil to lymphocyte ratio

The overall discriminatory ability in predicting the 1-year mortality was reasonable for TIMI risk score (c-statistic 0.841; 95% CI 0.796–0.886). The addition of HLI, dysglycemia, NLR, and anemia to the TIMI risk score significantly improved the c-statistic (from 0.841 to 0.876, *p* < 0.001; Fig. 1). The incremental improvement of the c-statistics was confirmed in each subgroup with Killip class I and class II–IV (Additional file 1: Figure S1). Table 4 shows reclassification for patients who died and those who did not, across 3 strata risks for 1-year mortality. When incorporating blood biomarkers, the risk reclassification was more accurate in 265 (27.0%) and less accurate in 66 (6.7%) patients, among a total of 982 patients of the Mortality (−) group (net improvement, 20.3%). However, addition of blood biomarkers to the TIMI risk score did not improve the reclassification of the Mortality (+) group (net improvement, 0.0%). Overall the NRI and IDI for predicting 1-year mortality using blood biomarkers plus TIMI risk score were 0.203 (95% CI 0.130–0.275; *p* < 0.001) and 0.089 (95% CI 0.060–0.119; *p* < 0.001), respectively.

**Table 4:** Reclassification among patient who died and those who did not die at 1-year after ST-segment elevation myocardial infarction using the routinely measured blood biomarkers over the TIMI risk score

1-year mortality	Model using blood biomarkers^†^ plus TIMI risk score			
	Low risk (< 1%)	Moderate risk (1–5%)	High risk (> 5%)	Total
Model using TIMI risk score				
No events				
Low risk (< 1%)	176 (88.44)	23 (11.56)	0 (0)	199
Moderate risk (1–5%)	164 (37.88)	226 (52.19)	43 (9.93)	4331
High risk (> 5%)	0 (0)	101 (28.86)	249 (71.14)	350
Total	340	350	292	982
Events				
Low risk (< 1%)	1 (100.0)	0 (0)	0 (0)	1
Moderate risk (1–5%)	1 (12.50)	4 (50.0)	3 (37.50)	8
High risk (> 5%)	0 (0)	2 (3.03)	64 (96.97)	66
Total	2	6	67	75
NRI = 0.203 (95% CI 0.130–0.275) IDI = 0.089 (95% CI 0.060–0.119)				

*NRI* net reclassification improvement, *IDI* integrated discrimination improvement

^†^The blood biomarkers included hypoxic liver injury, dysglycemia, anemia, and high neutrophil to lymphocyte ratio

## Discussion

The main findings of this study include: (1) HLI, dysglycemia, high NLR, and anemia were independently associated with the risk of 1-year mortality after primary PCI in patients with STEMI and (2) addition of these blood biomarkers to the TIMI risk score for STEMI significantly improved the discriminatory performance. To our knowledge, this is the first study to report that integrating blood biomarkers routinely measured in the emergency room with a conventional risk score incrementally improves the accuracy of 1-year mortality prediction in STEMI.

Many biomarkers are routinely measured at the time of presentation to monitor the general health status of a patient, including liver function, glucose metabolism, presence of anemia, or inflammation status [13]. These biomarkers are not commonly used for risk stratification in STEMI. In addition, conventional risk scoring systems for STEMI do not include routinely measured biomarkers as predictors, except for serum creatinine or hemoglobin. Previous studies have revealed that many biomarkers, such as serum transaminases, glucose, or NLR, are independently associated with poor outcomes in STEMI [3, 5, 6, 9, 14, 15]. However, there is little data that comprehensively evaluates the prognostic impacts of routinely measured blood biomarkers and the incremental value of combining these with a conventional risk score. In this study, we tried to integrate routine blood biomarkers for early risk stratification in STEMI patients. Early in the study, we considered serum creatinine, alkaline phosphatase, mean platelet volume, and creatine kinase-myocardial band isoenzyme as prognosticator. However, these biomarkers were not independent predictors for 1-year mortality. Finally, we included four biomarkers including HLI, dysglycemia, anemia, and high NLR among routinely measured blood tests on admission. In this respect, our model, which adds four predictors (HLI, dysglycemia, high NLR, and anemia) determined from routine blood tests on admission to an established TIMI risk score, had a substantial incremental impact on the discriminatory power. In particular, our combined model improved risk reclassification in patients without mortality, suggesting a more accurate capacity to reliably identify patients with low risk for mortality. This may help to select low-risk patients for early hospital discharge. A study of a prospective cohort, consisting of nearly 2000 MI patients, reported that adding several routine biomarkers returned a significantly better prediction of mortality or recurrent MI within 6 months than the GRACE risk score [16]. This study used 9 biomarkers as continuous variables, including urea, sodium, potassium, alkaline phosphatase, low-density lipoprotein cholesterol, glucose, hemoglobin, and C-reactive protein. It is difficult in practice to apply an additional biomarker as a continuous variable to a conventional risk scoring system at the bedside. Therefore, we divided biomarkers into binary variables, according to cut-off values that were previously determined or statistically calculated. This is a more practical approach for risk stratification at the time of presentation, without the aid of a computer. Yanish et al. [17] also developed a laboratory stratification model from the combination of routine blood tests to predict in-hospital mortality in patients with STEMI. Five variables (white blood cell, hemoglobin, C-reactive protein, creatinine, and glucose) were included in the laboratory model. The performance of this model was comparable to that of TIMI risk score and could be helpful in predicting in-hospital prognosis of STEMI patients by further subdividing the TIMI risk score. There are some differences between Yanish et al.’s study and ours. We focused 1-year mortality as an endpoint and integrated routine blood biomarkers into TIMI risk score, just not subclassifying the conventional risk score. In addition, HLI and NLR, recently emerged as prognostic biomarkers, included for our risk model.

It has been previously reported that each individual component of our risk model is significantly related with a high risk of adverse events. Recently, there have been a few reports stating the independent association between serum transaminases and cardiovascular outcomes in patients with STEMI [5, 18, 19]. The HLI, defined as increase in serum transaminases more than two times the upper limit of normal, was associated with high risk of mortality and major adverse cardiovascular events after primary PCI in STEMI [5]. Of note, Moon et al. [5] reported that HLI was closely correlated with left ventricular systolic dysfunction after PCI. Furthermore, admission and peak levels of serum transaminases were significantly related with infarct size, left ventricular ejection fraction, and the presence of microvascular damage in a study using cardiac magnetic resonance imaging [19]. The presence of HLI on admission may be a very sensitive marker suggesting significant left ventricular systolic dysfunction before primary PCI.

Previous studies have described the relationship between hyperglycemia on admission and poor outcomes in acute MI, regardless of the diabetic status [14, 20]. Hyperglycemia might aggravate organ damage by inflammation, endothelial dysfunction, increasing oxidative stress, and coagulation [21–23]. Hypoglycemia on admission has also been related to higher mortality, suggesting a U-shaped relationship between glucose levels and poor prognosis [15]. A plausible mechanism is that hypoglycemia increases the risk of arrhythmia, through changes in autonomic activity and effects on repolarization [24]. Our group also reported that dysglycemia with the same definition as that of the present study, was an independent predictor of in-hospital death in STEMI [6].

Anemia is associated with an increased risk for morbidity and mortality in acute MI [3, 9]. Anemia can potentially worsen myocardial ischemic insult by decreasing the oxygen content of blood delivered to the stressed heart and increasing myocardial oxygen demand and cardiac output for adequate systemic oxygen perfusion [25, 26]. Anemic patients have a higher prevalence of baseline comorbidities and receive less guideline-based pharmacological treatments and reperfusion therapy than non-anemic patients [27]. Anemia on admission is also associated with an increased risk of contrast-induced nephropathy after primary PCI [28]. Among the established risk scoring models in STEMI, only the CADILLAC (the Controlled Abciximab and Device Investigation to Lower Late Angioplasty Complications) risk score includes anemia as a predictor of mortality [29].

Finally, the NLR has recently emerged as a potential prognostic biomarker for various cardiovascular diseases [7, 8]. A high NLR has been associated with an increased risk of short- and long-term mortality after primary PCI in patients with STEMI [30, 31]. The NLR is an indicator of systemic inflammation, which plays a role in the repair and remodeling of infarcted heart tissue [32]. A high NLR (> 4.25) could predict adverse left ventricular remodeling 6 months after anterior STEMI [33]. Among patients with acute MI who underwent PCI, a high post-PCI NLR was related with the extent of myocardial infarction, as measured by cardiac magnetic resonance imaging [4]. In the present study, the best cut-off value of NLR to predict 1-year mortality was 4.3, which was consistent with that in previous studies [30, 31]. A high NLR was the most powerful predictor of 1-year mortality among the biomarkers routinely measured in blood tests.

The combination of HLI, anemia, dysglycemia, and NLR may represent an additional biochemical marker for indicating the severity and extent of myocardial damage, as well as the presence of adverse cardiac remodeling. This information can enhance other conventional risk predictors, such as clinical presentation, left ventricular systolic function, and atherosclerotic burden. The information on HLI, anemia, dysglycemia and NLR is typically taken from the very first routine blood test at the time of presentation, even before PCI. They are readily available and not costly. Thus, our study suggests that the combination of HLI, anemia, dysglycemia, and NLR with a traditional risk score model may provide valuable information for very early risk stratification in patients with STEMI undergoing primary PCI.

There are several limitations in this study. First, as an observational study, it is possible that residual confounding factors not adjusted by multivariate analysis were partially responsible for the present findings. Second, it is not conclusive that elevated serum transaminases were solely associated with hypoxic liver damage. In addition, the cut-off value of HLI have not been fully verified. Third, we had to calculate the best cut-off value of NLR, which may raise statistical inaccuracy. Fourth, the cut-off value of dysglycemia was determined based on our previous study, which was not validated in an external cohort. Finally, we did not externally validate our findings. Further studies are required to validate our findings externally and prospectively.

## Conclusions

The addition of routine blood biomarkers including HLI, dysglycemia, anemia, and high NLR to the TIMI risk score for STEMI significantly improved the discriminatory performance to predict 1-year mortality. Therefore, these biomarkers may be useful for very early risk stratification in patients with STEMI receiving primary PCI.

## Supplementary information

**Additional file 1**. Fig. S1. Receiver operating curves for the predicted probabilities of selected risk scores before (blue line) and after (red line) the addition of four biomarkers to the conventional TIMI risk score in each subgroup with Killip class I (A) and class II-IV (B). †The added biomarkers included hypoxic liver injury, dysglycemia, anemia and high neutrophil to lymphocyte ratio.

## Data Availability

The datasets used and/or analysed during the current study are available from the corresponding author on reasonable request.

## Abbreviations

NSTEMI: Non-ST-segment elevation myocardial infarction
STEMI: ST-segment elevation myocardial infarction
TIMI: Thrombolysis in myocardial infarction
GRACE: Global Registry of Acute Coronary Events
MI: Myocardial infarction
HLI: Hypoxic liver injury
NLR: Neutrophil to lymphocyte ratio
PCI: Percutaneous coronary intervention
ROC: Receiver operating characteristics
AUC: Area under the curve
HR: Hazard ratio
IDI: Integrated discrimination index
NRI: Net reclassification index
