# Supplementary material for: Addition of routine blood biomarkers to TIMI risk score improves predictive performance of 1-year mortality in patients with ST-segment elevation myocardial infarction
Source: BMC Cardiovasc Disord. 2020 Nov 18;20:486. doi: 10.1186/s12872-020-01777-7 (PMC7672980; doi:10.1186/s12872-020-01777-7)
Supplement: Supplementary file 1 — Additional file 1. Fig. S1. Receiver operating curves for the predicted probabilities of selected risk scores before (blue line) and after (red line) the addition of four biomarkers to the conventional TIMI risk score in each subgroup with Killip class I (A) and class II-IV (B). †The added biomarkers included hypoxic liver injury, dysglycemia, anemia and high neutrophil to lymphocyte ratio. [file 12872_2020_1777_MOESM1_ESM.pptx]

## Slide 1
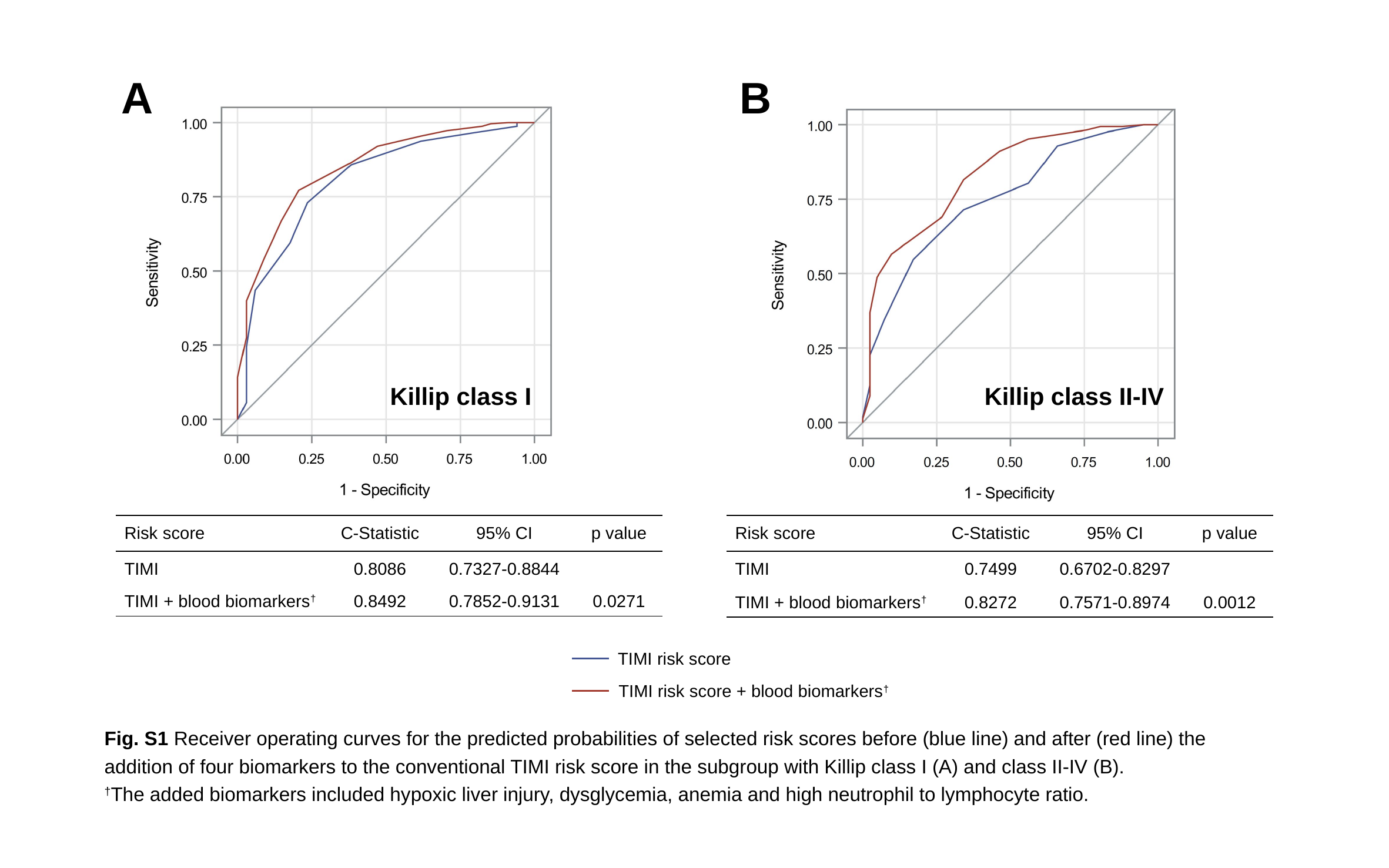

A
B
Killip class I
Killip class II-IV
| Risk score | C-Statistic | 95% CI | p value |
| --- | --- | --- | --- |
| TIMI | 0.8086 | 0.7327-0.8844 | |
| TIMI + blood biomarkers† | 0.8492 | 0.7852-0.9131 | 0.0271 |
| Risk score | C-Statistic | 95% CI | p value |
| --- | --- | --- | --- |
| TIMI | 0.7499 | 0.6702-0.8297 | |
| TIMI + blood biomarkers† | 0.8272 | 0.7571-0.8974 | 0.0012 |
TIMI risk score
TIMI risk score + blood biomarkers†
Fig. S1 Receiver operating curves for the predicted probabilities of selected risk scores before (blue line) and after (red line) the addition of four biomarkers to the conventional TIMI risk score in the subgroup with Killip class I (A) and class II-IV (B).
†The added biomarkers included hypoxic liver injury, dysglycemia, anemia and high neutrophil to lymphocyte ratio.
